# Effect of infant viral respiratory disease on childhood asthma in a non‐industrialized setting

**DOI:** 10.1002/clt2.12291

**Published:** 2023-08-08

**Authors:** Jessica Atwell, Martha Chico, Maritza Vaca, Andrea Arévalo‐Cortes, Ruth Karron, Philip J. Cooper

**Affiliations:** ^1^ Johns Hopkins Bloomberg School of Public Health Baltimore Maryland USA; ^2^ Fundacion Ecuatoriana Para la Investigacion en Salud Quito Ecuador; ^3^ Escuela de Medicina Universidad Internacional del Ecuador Quito Ecuador; ^4^ Institute of Infection and Immunity St George's University of London London UK

**Keywords:** asthma, helminths, rhinoconjunctivitis, viral infections, wheeze

## Abstract

**Background:**

There are limited data from non‐industrialized settings on the effects of early life viral respiratory disease on childhood respiratory illness. We followed a birth cohort in tropical Ecuador to understand how early viral respiratory disease, in the context of exposures affecting airway inflammation including ascariasis, affect wheezing illness, asthma, and rhinoconjunctivitis in later childhood.

**Methods:**

A surveillance cohort nested within a birth cohort was monitored for respiratory infections during the first 2 years in rural Ecuador and followed for 8 years for the development of wheeze and rhinoconjunctivitis. Nasal swabs were examined for viruses by polymerase chain reaction and respiratory symptom data on recent wheeze and rhinoconjunctivitis were collected by periodic questionnaires at 3, 5, and 8 years. Stools from pregnant mothers and periodically from children aged 2 years were examined microscopically for soil‐transmitted helminths. Atopy was measured by allergen skin prick testing at 2 years. Spirometry, fractional exhaled nitric oxide measurement, and nasal washes were performed at 8 years. Associations between clinically significant respiratory disease (CSRD) and wheezing or rhinoconjunctivitis at 3, 5, and 8 years were estimated using multivariable logistic regression.

**Results:**

Four hundred and twenty six children were followed of which 67.7% had at least one CSRD episode; 12% had respiratory syncytial virus (RSV)+CSRD and 36% had rhinovirus (RHV)+CSRD. All‐cause CSRD was associated with increased wheeze at 3 (OR 2.33 [95% confidence intervals (CI) 1.23–4.40]) and 5 (OR: 2.12 [95% CI 1.12–4.01]) years. RHV+CSRD was more strongly associated with wheeze at 3 years in STH‐infected (STH‐infected [OR 13.41, 95% CI 1.56–115.64] vs. uninfected [OR 1.68, 95% CI 0.73–3.84]) and SPT+ (SPT+ [OR 9.42, 95% CI 1.88–47.15] versus SPT‐ [OR 1.92, 95% CI 0.84–4.38]) children. No associations were observed between CSRD and rhinoconjunctivitis.

**Discussion:**

CSRD was significantly associated with childhood wheeze with stronger associations observed for RHV+CSRD in SPT+ and STH‐infected children.

## 1 BACKGROUND

Respiratory syncytial virus (RSV) is the leading viral cause of severe acute lower respiratory infections (LRI) in infants and children worldwide, causing up to 80% of bronchiolitis and an estimated >3.6 million hospitalizations and 84,500–125,200 deaths annually among children below 5 years of age.^1^, ^2^ Rhinovirus (RHV) is responsible for up to 20%–40% of medically attended acute wheezing and bronchiolitis in infancy, and is a leading cause of LRI outside the RSV season. Viral infections in infancy, particularly early LRI due to RSV and RHV, have been associated with recurrent wheezing and asthma in children.^3^, ^4^, ^5^, ^6^ Important questions remain about the directionality of the associations between viral LRI in infants and airway disease in children and potential interactions with atopy.^5^, ^6^, ^7^, ^8^ If causal associations do exist, the prevention of early viral LRI may reduce wheezing illness in early childhood and/or asthma in older children.

Although numerous studies over the last 30 years have evaluated these relationships,^9^, ^10^, ^11^, ^12^ few have been conducted in resource‐limited rural settings in low and middle‐income countries where the burden of acute LRI is considered to be disproportionately high and a number of environmental factors might interact with early viral LRI to alter long‐term wheezing and airway outcomes.^1^

The hygiene hypothesis has postulated a protective role of common early childhood infections^13^ and exposure to a wide diversity of microbes^14^ against allergy and asthma. In recent years, there has been interest in the role of early life helminth infections in modulating the development of allergy or atopy and wheezing illness in childhood.^15^, ^16^, ^17^, ^18^ Soil‐transmitted helminths (STH), primarily *Ascaris lumbricoides* and *Trichuris trichiura*, are estimated to infect over 1 billion humans worldwide.^19^ A severe Th2‐inflammatory response^20^ associated with severe pulmonary disease^21^ can be induced in mice experimentally infected with *Ascaris*, while potentially severe obstructive airway disease has been observed in humans with evidence of sporadic^22^ or seasonal^23^ exposures to *Ascaris*.

Early life exposure to STH parasites could modify the effects of early viral LRI on wheezing illness and asthma in later childhood through effects on airway inflammation. We followed a birth cohort in a tropical, rural region of Ecuador to investigate associations between clinically significant respiratory illness (CSRD) in the first 2 years of life and pulmonary outcomes at 3, 5, and 8 years in the context of endemic STH infections. We explored whether associations between CSRD and later respiratory outcomes might be modified by antenatal and early childhood exposure to STH parasites, and by early life endotoxin exposure and atopy at 2 years.

## 2 METHODS

### 2.1 Study population

This study was nested within the ECUAVIDA birth cohort, a study of 2404 children born between 2006 and 2009 in the district of Quinindé, Esmeraldas Province, in a rural tropical coastal region in northeastern Ecuador. ECUAVIDA was designed to evaluate the effects of a wide range of infectious and non‐infectious factors on development of atopy and allergic disease to 8 years.^24^ The cohort was recruited at the public hospital in the town of Quinindé, the only hospital serving the district. All newborns born at the hospital during the recruitment period were eligible for recruitment according to the following criteria: (i) healthy baby aged less than 14 days; (ii) residence in the district of Quinindé; (iii) accessible household; and (iv) maternal stool sample during pregnancy. The present analysis focused on a subset of 504 children enrolled consecutively during October 2008 and December 2009. Detailed active respiratory disease surveillance during the first 2 years of life was performed on 426 of these children who lived within a 10 km radius of the town of Quinindé. Follow‐up at 8 years was completed between October 2016 and December 2017. Further details regarding data and specimen collection, testing and follow‐up are provided elsewhere.^24^

### 2.2 Routine procedures

Information on demographic, socioeconomic and household information, and respiratory symptoms were obtained using a questionnaire administered to the child's mother during routine visits around the time of birth of the child and at 3, 5, and 8 years. Relevant data were collected by questionnaire and physical examinations during clinic and home visits.^24^

### 2.3 Atopy

Allergen skin prick testing (SPT) was performed at 2 years for a panel of nine allergens (house dust mites, cockroach, dog, cat, fungi, grass pollens, egg, milk, and peanut) as described.^25^ A positive test was a mean wheal diameter ≥2 mm greater than the negative saline control. SPT positivity was defined as reactivity to any allergen.

### 2.4 Stool analyses

STH infections were detected by microscopy of stool samples collected from the mother during the third trimester of pregnancy and from children at 3, 7, 13, and 24 months using a combination of direct saline smears, Kato‐Katz, formol‐ether concentrations, and carbon coproculture.^24^

### 2.5 Airways function and inflammation at 8 years

Pulmonary function was measured using a MicroLoop spirometer (CareFusion) before and after 200 μg salbutamol. A positive test for airway reactivity was an increase in FEV1 of ≥12%. Global Lung Function Initiative reference values were used to calculate *z*‐scores for FEV1, FVC, and FEV1/FVC, adjusting for age, sex, height, and ethnicity.^26^ Fractional exhaled nitric oxide was measured in parts per billion (ppb) using NObreath (Bedfont Scientific). Values greater than 35 ppb were considered elevated.^27^ Nasal wash samples were collected and prepared as described^28^: slides with ≥5% eosinophils were considered eosinophilic.

### 2.6 Nasopharyngeal swabs

Once respiratory surveillance was initiated, nasopharyngeal (NP) swabs were collected whenever children had symptomatic respiratory illness through a combination of active and passive surveillance through clinic and home visits for evaluation of acute respiratory illnesses. Routine telephone calls were made twice weekly to mothers to determine whether the child in the surveillance sample had respiratory symptoms, and medical records of visits to a dedicated cohort pediatric clinic were reviewed. Swabs were stored at −80°C and tested using multiplex rRT‐polymerase chain reaction (PCR) (FTD Respiratory pathogens 21, Fast‐Track Diagnostics, Luxembourg) for a range of respiratory pathogens including RHV and RSV.

### 2.7 Endotoxin

Mattress samples for the measurement of endotoxin were obtained from the sleeping place of each child at 13 months using 1200W electric vacuum cleaners (Electrolux) and nylon filters (Indoor Biotechnologies, Inc.) to aspirate an uncovered area of 0.25 m^2^ over 2 min. Dust samples were stored at −20°C prior to shipment for analysis of endotoxin levels (EU/mL) using the kinetic Limulus Amoebocyte Lysate assay (Indoor Biotechnologies).

### 2.8 Definitions

Respiratory outcomes were defined: (1) CSRD—any respiratory illness with rales, wheezing, tachypnea, subcostal retraction, or nasal flaring detected on physical examination and/or a diagnosis of bronchiolitis, bronchitis, pneumonia; (2) recent wheeze—parental report of wheeze during the previous 12 months; (3) recurrent wheezing—parental report of wheezing on two or more occasions during the previous 12 months; (4) asthma—a parental report of wheeze over the previous 3 years at 8 years of age, and parentally reported wheeze up to 5 years or physician‐diagnosed asthma at any time, or both; and (5) recent rhinoconjunctivitis—a parental report during the previous 12 months of relevant nasal symptoms in the absence of a cold and accompanied by itchy watery eyes.^29^

### 2.9 Bias

Stringent procedures to prevent losses to follow‐up or incomplete data and sampling were implemented, including regular contacts by phone and home visits and maintaining a registry of neighborhood contacts. Reliance on maternal recall for the collection of questionnaire data could be subject to bias because of differential recall depending on maternal age and educational level. Data on a wide variety of relevant risk factors were collected to control for potential confounding. Exposures were measured using objective and standardized protocols (including laboratory methods for respiratory viruses, STH infections, and endotoxin) which will have minimized measurement bias. Data on outcomes were collected blind to exposure status. The original cohort study (of 2404 newborns) was designed to measure the effect of childhood STH infections on allergic outcomes and not for the effects of CSRD on wheeze and rhinoconjunctivitis. The sample size for this sub‐sample of the cohort selected for respiratory infection surveillance was restricted by logistical and cost considerations. Our analysis of this sub‐sample of the cohort with respiratory infection surveillance had limited power for detection of associations between infrequent exposures and or outcomes.

### 2.10 Statistical analyses

The primary analysis examined associations between CSRD and wheeze at 3, 5, and 8 years. Secondary analyses addressed associations between CSRD and other respiratory outcomes (recurrent wheeze, asthma diagnosis, and markers of airways function and inflammation). Bivariate and multivariable logistic regression analyses were done to estimate associations with CSRD and potential confounders with outcomes. Potential confounders considered in the analyses are shown in Table 1. A socio‐economic status index was created using principal component analysis as described.^17^ Potential confounders in any of the bivariate analyses with *p* < 0.10 were kept in the final models using the same set of confounders to adjust all models. All multivariable models were controlled for sex. Potential a priori effect modifiers (maternal [any vs. none] and childhood [any vs. none during first 2 years of life] STH infections, and endotoxin exposure [>median vs. ≤ median of 18 EU/mL]) were evaluated using likelihood ratio tests. Significance was set at *α* = 0.05. Population fractions of CSRD attributable to individual pathogens were estimated as described.^30^ Sensitivity analyses for uncertainty in estimates were explored including missing data assumed to be at random, and results did not qualitatively change the findings. All statistical analyses were performed using Stata 11 (Statacorp).

**TABLE 1:** Associations between clinically significant respiratory disease (CSRD), potential confounders, and childhood wheeze between 3 and 8 years of age in a sample of 424 children surveillance cohort.

Characteristics	*N* (%)	Wheeze at 3 years (*n* = 405)			Wheeze at 5 years (*n* = 381)			Wheeze at 8 years (*n* = 363)		
		*N* (%)	OR (95% CI)	*p* Value	*N* (%)	OR (95% CI)	*p* Value	*N* (%)	OR (95% CI)	*p* Value
Individual										
Sex										
Male	200 (47.3)	38 (19.8)	1		33 (18.4)	1		14 (8.5)	1	
Female	224 (52.7)	35 (16.4)	0.80 (0.48–1.32)	0.380	38 (18.8)	1.03 (0.61–1.72)	0.925	9 (4.6)	0.51 (0.22–1.22)	0.131
Breastfeeding										
<6 months	51 (12.4)	4 (7.8)	1		12 (24.5)	1		4 (8.5)	1	
>6 months	360 (87.6)	69 (19.7)	**2.89 (1.01–8.28**)	**0.049**	59 (18.1)	0.68 (0.34–1.39)	0.289	19 (6.1)	0.70 (0.23–2.15)	0.529
Allergen SPT at 2 years										
No	336 (82.1)	54 (16.4)	1		57 (18.3)	1		16 (5.4)	1	
Yes	73 (20.9)	19 (27.1)	**1.90 (1.04–3.46)**	**0.037**	14 (22.6)	1.30 (0.67–2.52)	0.437	7 (11.7)	1.53 (0.90–2.61)	0.118
Any STH to 2 years										
No	332 (79.1)	55 (17.4)	1		57 (19.1)	1		20 (7.0)	1	
Yes	88 (20.9)	18 (21.2)	1.27 (0.70–2.31)	0.425	14 (17.7)	0.91 (0.48–1.74)	0.786	3 (4.0)	0.55 (0.16–1.92)	0.351
CSRD to 2 years										
No	137 (32.3)	14 (10.7)	1		14 (11.4)	1		3 (2.6)	1	
Yes										
All	287 (67.7)	59 (21.5)	**2.29 (1.23–4.28)**	**0.009**	57 (22.1)	**2.21 (1.18–4.14)**	**0.014**	20 (8.1)	3.27 (0.95–11.25)	0.060
RSV+	18 (4.3)	3 (18.8)	1.93 (0.49–7.61)	0.348	3 (18.8)	1.80 (0.46–7.09)	0.403	2 (15.4)	**6.79 (1.02–45.08)**	**0.047**
RHV+	119 (28.0)	26 (22.4)	**2.41 (1.19–4.89)**	**0.014**	22 (20.2)	1.97 (0.95–4.07)	0.068	7 (6.5)	2.61 (0.66–10.38)	0.172
RSV+/RHV+	32 (7.5)	9 (30.0)	**3.58 (1.37–9.33)**	**0.009**	9 (31.0)	**3.50 (1.34–9.18)**	**0.011**	3 (12.0)	5.09 (0.96–26.89)	0.055
RSV‐RHV‐	63 (14.9)	12 (19.4)	2.01 (0.87–4.64)	0.104	12 (21.4)	2.12 (0.91–4.95)	0.081	5 (8.9)	3.66 (0.84–15.91)	0.083
No PCR result	55 (13.0)	9 (18.0)	1.83 (0.74–4.56)	0.191	11 (22.9)	2.31 (0.97–5.54)	0.060	3 (6.4)	2.55 (0.49–13.09)	0.264
Maternal										
Educational status										
Illiterate	62 (14.6)	15 (25.9)	1		14 (26.9)	1		2 (4.1)	1	
Non‐illiterate	362 (35.4)	58 (16.7)	0.58 (0.30–1.10)	0.096	57 (17.3)	0.57 (0.29–1.12)	0.102	21 (6.7)	1.69 (0.38–7.42)	0.491
Ethnicity										
Afro‐Ecuadorian	128 (30.2)	21 (17.2)	1		21 (18.4)	1		9 (7.9)	1	
Non‐Afro‐Ecuadorian	196 (69.8)	52 (18.4)	1.08 (0.62–1.89)	0.780	55 (18.7)	1.02 (0.58–1.79)	0.944	14 (5.6)	0.70 (0.29–1.66)	0.412
Allergic symptoms										
No	412 (97.9)	70 (17.8)	1		70 (18.8)	1		21 (5.9)	1	
Yes	9 (2.1)	3 (33.3)	2.31 (0.57–9.48)	0.243	1 (16.7)	0.87 (0.10–7.53)	0.896	2 (28.6)	**6.34 (1.16–34.65)**	**0.033**
Maternal STH										
No	235 (56.1)	33 (14.6)	1		31 (14.8)	1		12 (6.0)	1	
Yes	184 (43.9)	37 (21.3)	1.58 (0.94–2.65)	0.084	37 (22.3)	1.66 (0.98–2.81)	0.061	11 (6.9)	1.16 (0.50–2.70)	0.734
Household										
Socioeconomic level										
Low	117 (27.6)	24 (21.6)	1		16 (15.5)	1		6 (6.2)	1	
Medium	151 (35.6)	24 (16.7)	0.73 (0.39–1.36)	0.317	30 (22.2)	1.55 (0.79–3.04)	0.197	7 (5.3)	0.86 (0.28–2.63)	0.786
High	156 (36.8)	25 (16.7)	0.73 (0.39–1.35)	0.312	25 (17.5)	1.15 (0.58–2.29)	0.686	10 (7.4)	1.21 (0.43–3.46)	0.718
Overcrowding										
No	197 (46.5)	27 (14.6)	1		26 (14.9)	1		12 (7.3)	1	
Yes	227 (53.5)	46 (20.9)	1.55 (0.92–2.61)	0.101	45 (21.7)	1.58 (0.93–2.69)	0.091	11 (5.6)	0.75 (0.32–1.75)	0.505
Area of residence										
Urban	301 (71.0)	56 (19.5)	1		52 (19.3)	1		19 (7.5)	1	
Rural	123 (29.0)	17 (14.4)	0.69 (0.39–1.25)	0.266	19 (17.0)	0.85 (0.48–1.52)	0.589	4 (3.6)	0.46 (0.15–1.40)	0.173
Endotoxin (quartiles)										
1^st^ (3–12 EU/mg)	111 (28.3)	25 (23.6)	1		20 (20.2)	1		8 (8.6)	1	
2^nd^ (13–18 EU/mg)	86 (21.9)	9 (11.0)	**0.40 (0.18–0.91)**	**0.029**	10 (12.3)	0.56 (0.25–1.29)	0.174	1 (1.4)	0.15 (0.02–1.23)	0.077
3^rd^ (19–34 EU/mg)	98 (25.0)	18 (19.0)	0.76 (0.38–1.50)	0.424	19 (22.1)	1.12 (0.55–2.27)	0.753	7 (7.9)	0.90 (0.31–2.61)	0.857
4^th^ (35–1393 EU/mg)	97 (24.7)	17 (18.5)	0.73 (0.37–1.47)	0.382	17 (19.8)	0.97 (0.47–2.00)	0.941	6 (7.4)	0.85 (0.28–2.56)	0.770

*Note*: ORs and 95% confidence intervals (CI) estimated using logistic regression. Separate models were used to estimate associations with all CSRD and CSRD by respiratory virus subgroups. SPT, allergen skin prick test reactivity; STH, soil‐transmitted helminth infection; RSV, PCR+ for respiratory syncytial virus; RHV+, PCR+ for rhinovirus; RSV+/RHV+, PCR+ for both viruses; RSV‐/RHV‐, PCR‐ for both viruses. Household overcrowding defined by ≥3 persons/sleeping room. Missing: breastfeeding (13), allergen SPT at 2 years (15), any STH to 2 years (4), maternal allergic symptoms (4), maternal STH infections (5), and mattress endotoxin (34). Bold values denote statistical significance at the *p* < 0.05 level.

### 2.11 Ethics considerations

Study protocols were approved by ethics committees in Ecuador (Hospital Pedro Vicente Maldonado, Universidad San Francisco de Quito, and Universidad Internacional del Ecuador) and the UK (London School of Hygiene and Tropical Medicine). The study was registered as an observational study (ISRCTN41239086). Informed written consent was obtained from the child's mother and minor assent was obtained from the child after 8 years. Anthelmintic treatment was provided to mothers and children with positive stools for STH as recommended.^31^

## 3 RESULTS

### 3.1 Surveillance cohort participants

**FIGURE 1:**
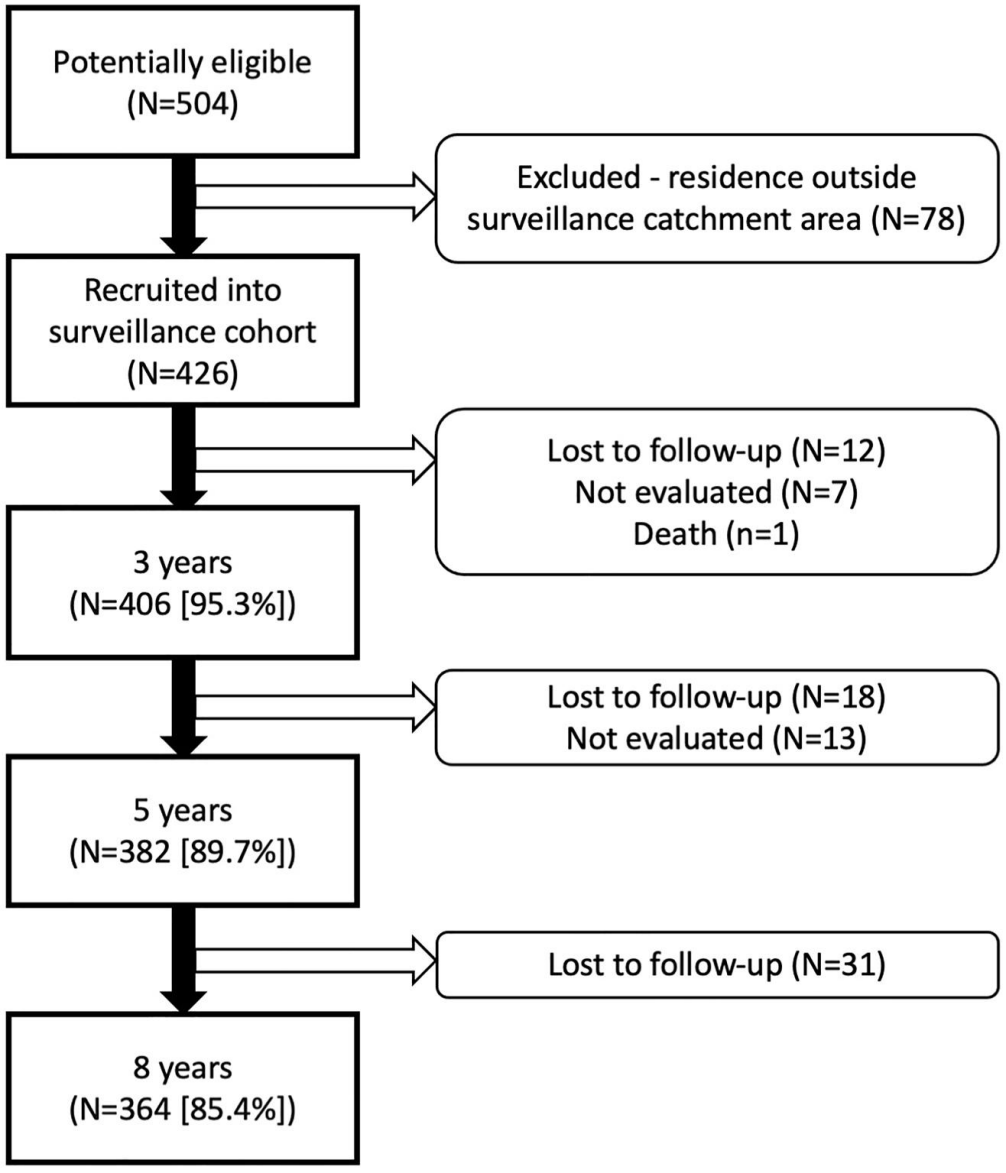
Participant flow of 426 children recruited into the surveillance cohort through follow‐up to 8 years of age and those included and excluded from the analysis.

As shown in Figure 1, 504 children in the ECUAVIDA cohort were considered for participation in surveillance; 78 were excluded because of accessibility (i.e. lived outside a 10 km radius of Quininde). Thus, 426 infants were included in surveillance. Follow‐up was over 85% at each of the follow‐up times from 3 to 8 years (Figure 1). Data on individual, maternal and household factors for 426 study participants are shown in Table 1. Births occurred between late 2008 and the end of 2009. One child died before one year of age of unknown cause. Baseline characteristics did not differ significantly between the children included in the analysis and those initially considered for participation (data not shown).

### 3.2 Viruses during first 2 years of life

**FIGURE 2:**
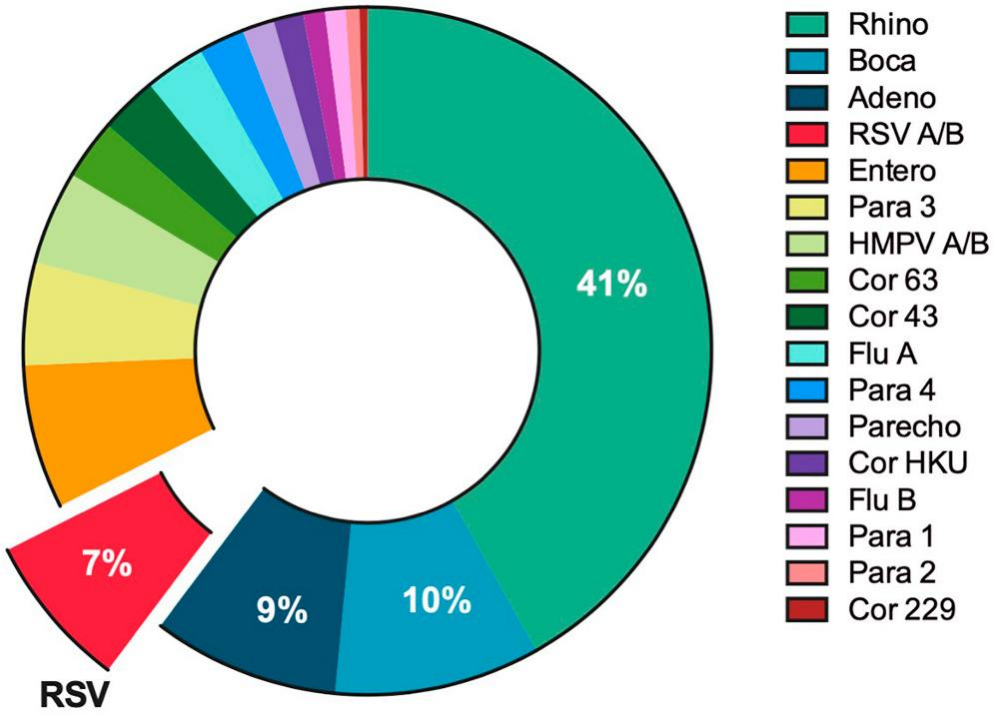
Viruses detected during the first 2 years of life in a surveillance cohort of 426 children. Data are for 1894 positive PCR reactions from 1809 swabs collected during the first 2 years of life. PCR, polymerase chain reaction.

Nasopharyngeal (NP) swab collection began in the January 2009 and continued through 2011 when the youngest enrolled children reached 2 years. Multiplex real‐time PCR was performed on 1635 swabs collected from 426 children; results are shown in Figure 2. RHV (41%) was most frequently detected followed by bocavirus (10%), adenovirus (9%), and RSV (7%). Only 84/1635 swabs (5%) were negative for all pathogens, while 375 (23%) swabs were positive for 1 pathogen, 619 (38%) were positive for 2, 391 (24%) were positive for 3, and 166 (10%) for ≥4 (maximum 7 pathogens). RSV was seasonal (from January to July) while rhinovirus (RHV) was detected year‐round. Analysis of the age of children in the follow‐up relative to the seasonality of RSV revealed that the number of children aged up to 6 months under surveillance when RSV was circulating was limited (*n* = 210, 42%), likely resulting in missing RSV in early infancy for those born during the 2009 RSV season.

### 3.3 Clinically significant respiratory disease (CSRD)

CSRD during the first 2 years of life was common affecting 287/426 (67.7%) children (Table 1) Among these, 32 (7.5%) had CSRD associated with positivity for both RSV+ and RHV+, 18 with RSV+ (4.3%) only, and 119 (28.0%) with RHV+ only. Children in the RSV+/RHV+ group included those with dual‐positivity or multiple episodes of single pathogen CSRD. RSV+ (OR 4.53, 95% confidence intervals [CI] 2.1–11.3, *p* < 0.001) and RHV+ (OR 2.46, 95% CI 1.77–3.42, *p* < 0.01) were significantly associated with CSRD. Population attributable fractions for CSRD associated with individual pathogens were 28% for RSV+, 4.3% for RHV+, and 7.5% for both pathogens. Approximately 2.4% of children were hospitalized for respiratory illness over this period.

### 3.4 Respiratory outcomes

Wheezing during the previous 12 months was observed in 18.0%, 18.6%, and 6.3% of children at 3, 5 and 8 years, respectively; for recurrent wheeze, the proportions at these time points were 10.6%, 11.3%, and 3.3%, respectively. Asthma was present in 9.1% of children at 8 years. Evidence of impaired lung function (<−1.64 *z* scores) by spirometry (out of 299 with valid tests) was observed in 11.4% for FEV1, 17.4% for FVC, and 2.0% for FEV1/FVC. Evidence of elevated FeNO (>35 ppb) was observed in 8.6% of children and nasal eosinophilia in 11.4%. Rhinoconjunctivitis during the previous 12 months was observed in 12.5%, 14.5%, and 9.1% of children at 3, 5 and 8 years, respectively.

### 3.5 Endotoxin, SPT, and STH infections

Median endotoxin levels in bed dust were 18 EU/mg (geometric mean 21.5; range 3–1394). SPT reactivity was observed in 17.9% of children at 2 years. STH infections were detected in 43.9% of mothers (26.0% had *A. lumbricoides* and 25.1% had *T. trichiura*). STH infections during the first 2 years of life were detected in 21.0% of children (16.0% with *A. lumbricoides* and 8.6% with *T. trichiura*).

### 3.6 Associations between CSRD and respiratory outcomes

Univariable associations between CSRD and individual, maternal, and household risk factors and recent wheeze at 3, 5 and 8 years of age are shown in Table 1 and multivariable associations are shown in Table 2. In multivariable analyses, all‐cause CSRD was significantly associated with recent wheeze at 3 (adj. OR 2.33, 95% CI 1.23–4.40, *p* = 0.009) and 5 years (adj. OR 2.12, 95% CI 1.12–4.01, *p* = 0.021). RHV+ CSRD was associated with recent wheeze at 3 years (adj. OR 2.44, 95% CI 1.19–5.00, *p* = 0.015) while RSV+/RHV+ CSRD was associated with wheeze at 3 (adj. OR 3.49, 95% CI 1.31–9.33, *p* = 0.013) and 5 (adj. OR 3.32, 95% CI 1.25–8.78, *p* = 0.016) years. Similar associations were observed between CSRD and recurrent wheeze at 3 (adj. OR 2.66, 95% CI 1.13–6.27, *p* = 0.025) and 5 years (adj. OR 2.44, 95% CI 1.04–5.71, *p* = 0.040). No significant associations were observed between CSRD and recent wheeze (Table 2), recurrent wheeze, or asthma at 8 years (data not shown). There were no associations between all‐cause CSRD or CSRD sub‐groups and elevated FeNO at 8 years (data not shown), while RHV+CSRD was associated with elevated nasal eosinophil counts (adj. OR 3.28, 95% CI 1.27–8.48, *p* = 0.014). Parameters of diminished airway function (FEV_1_, FVC, and FEV_1_/FVC <1.64 *z* scores) or evidence of reversibility were not associated with all‐cause CSRD or CSRD sub‐groups (data not shown). Analysis of the subsample of 210 children born after July 2009, and aged 3–6 months during the 2010 RSV season showed comparable associations between RSV+CSRD and wheeze at 3 (adj. OR 1.62, 95% CI 0.27–9.73), 5 (adj. OR 4.94, 95% CI 0.37–66.30) and 8 years (adj. OR 4.76, 95% CI 0.31–73.69). Univariate associations between CSRD and CSRD sub‐groups and rhinoconjunctivitis are shown in Table S1. No significant associations were observed between CSRD (3 years—OR 0.82, 95% CI 0.44–1.53, *p* = 0.536; 5 years—OR 1.08, 95% CI 0.58–2.01, *p* = 0.803; and 8 years—OR 1.50, 95% CI 0.65–3.432, *p* = 0.338) or CSRD sub‐groups and rhinoconjunctivitis at 3, 5, and 8 years.

**TABLE 2:** Multivariable associations between clinically significant respiratory disease (CSRD) and childhood wheeze between 3 and 8 years of age.

CSRD group	Wheeze at 3 years		Wheeze at 5 years		Wheeze at 8 years	
	OR (95% CI)	*p* Value	OR (95% CI)	*p* Value	OR (95% CI)	*p* Value
CSRD to 2 years						
No	1		1		1	
Yes						
All CSRD	**2.33 (1.23–4.40)**	**0.009**	**2.12 (1.12–4.01)**	**0.021**	2.88 (0.82–10.10)	0.099
RSV+	1.98 (0.48–8.17)	0.346	2.19 (0.52–9.14)	0.282	4.47 (0.55–35.99)	0.160
RHV+	**2.44 (1.19–5.00)**	**0.015**	1.87 (0.90–3.89)	0.094	2.48 (0.61–9.96)	0.202
RSV+/RHV+	**3.49 (1.31–9.33)**	**0.013**	**3.32 (1.25–8.78)**	**0.016**	3.86 (0.68–21.92)	0.128
RSV‐RHV‐	1.98 (0.84–4.66)	0.120	2.01 (0.86–4.73)	0.109	3.40 (0.77–15.04)	0.106
No PCR result	1.93 (0.76–4.90)	0.267	2.15 (0.89–5.21)	0.091	2.21 (0.41–11.76)	0.353

*Note*: ORs and 95% confidence intervals (CI) estimated using logistic regression and adjusted for sex, breastfeeding, SPT at 2 years, and maternal allergy. Separate models were used to estimate associations with all CSRD and CSRD by respiratory virus subgroups. SPT, allergen skin prick test reactivity; RSV, PCR+ for respiratory syncytial virus; RHV+, PCR+ for rhinovirus; RSV+/RHV+, PCR+ for both viruses; RSV‐/RHV‐, PCR‐ for both viruses. Bold values denote statistical significance at the *p* < 0.05 level.

### 3.7 Effect modification by endotoxin level, SPT, or STH infections

Multivariable associations between CSRD and recent wheeze at 3 years stratified by potential effect modifiers (i.e. maternal and childhood STH, endotoxin levels, and SPT+ at 2 years) are shown in Table 3. There was no statistical evidence of effect modification by any of these exposures on CSRD‐recent wheeze associations. Analysis of associations between CSRD sub‐groups and recent wheeze at 3 years stratified by the same variables are shown in Table 4. None of the atopic children or those infected with STH had wheeze‐associated RSV+ CSRD. There was some evidence for stronger associations between RHV‐positive CSRD (i.e. RHV+CSRD [adj. OR 9.42, 95% CI 1.88–47.15], and any RHV+ sub‐groups [adj. OR 8.86, 95% CI 1.88–41.77]) among atopic compared to non‐atopic children while the reverse was seen for the (any) RSV+ sub‐group (adj. OR 3.25, 95% CI 1.20–8.76). Associations between RHV+CSRD and wheeze was stronger among STH‐infected children.

**TABLE 3:** Analysis of potential effect modification by key environmental exposures and individual characteristics on associations between clinically significant respiratory disease and wheeze at 3, 5, and 8 years of age.

Variable	Wheeze at 3 years				Wheeze at 5 years				Wheeze at 8 years			
	*N*/%	OR (95% CI)	*p* Value	Interaction *p* value	*N*/%	OR (95% CI)	*p* Value	Interaction *p* value	*N*/%	OR (95% CI)	*p* Value	Interaction *p* value
Endotoxin												
Low endotoxin												
CSRD												
No	70/11.4%	1			68/10.3%	1			62/3.2%	1		
Yes	137/21.2%	2.04 (0.86–4.85)	0.108	0.621	130/20.0%	2.04 (0.82–5.08)	0.124	0.512	122/6.6%	1.62 (0.31–8.44)	0.566	0.313
High endotoxin												
CSRD												
No	76/9.2%	1			70/14.3%	1			67/1.5%	1		
Yes	152/21.7%	**2.71 (1.11–6.60)**	**0.029**		143/23.1%	1.54 (0.69–3.41)	0.287		140/9.3%	6.60 (0.83–52.64)	0.075	
SPT at 2 years												
No SPT												
CSRD												
No	110/10.0%	1			14/13.5%	1			97/1.0%	1		
Yes	225/19.1%	**2.14 (1.05–4.37)**	**0.037**	0.600	215/20.0%	1.55 (0.80–3.02)	0.195	‐‐‐‐‐‐‐‐	206/7.3%	6.29 (0.81–48.89)	0.079	0.134
SPT												
CSRD												
No	25/12.0%	1			23/0%				21/9.5%	1		
Yes	51/31.4%	**4.19 (1.02–17.25)**	**0.047**		47/29.8%	‐‐‐‐‐‐‐‐‐‐‐‐‐‐‐‐‐‐‐‐‐	‐‐‐‐‐‐‐		45/11.1%	1.19 (0.18–7.95)	0.858	
Maternal STH												
No STH												
CSRD												
No	90/7.8%	1			82/11.0%	1			77/2.6%	1		
Yes	141/20.6%	**3.36 (1.38–8.17)**	**0.008**	0.192	133/18.8%	1.85 (0.81–4.27)	0.146	0.507	127/7.9%	3.30 (0.70–15.65)	0.133	0.928
STH												
CSRD												
No	42/19.1%	1			42/11.9%	1			39/2.6%	1		
Yes	137/23.4%	1.34 (0.55–3.26)	0.516		129/27.1%	**2.80 (1.00–7.83)**	**0.049**		125/8.0%	2.36 (0.28–20.0)	0.432	
Childhood STH												
No STH												
CSRD												
No	112/11.6%	1			106/11.3%	**1**			99/3.0%	1		
Yes	208/20.2%	**2.09 (1.05–4.15)**	**0.035**	0.230	196/23.0%	**2.27 (1.14–4.55)**	**0.020**	0.751	189/9.0%	3.24 (0.90–11.70)	0.073	‐‐‐‐‐‐
STH												
CSRD												
No	23/4.4%	1			20/10.0%	1			18/0%			
Yes	66/25.8%	7.57 (0.94–6.13)	0.058		62/19.4%	2.12 (0.42–10.74)	0.362		59/5.1%	‐‐‐‐‐‐‐‐‐‐‐‐‐‐‐‐‐‐‐‐‐‐‐	‐‐‐‐‐‐‐	

*Note*: Exposures/characteristics studied were endotoxin levels (> median vs. ≤ median of 18 EU/mL), allergen skin prick test reactivity (SPT) at 2 years of age, maternal soil‐transmitted helminth infections (STH) and childhood STH infections during first 2 years of life. Shown are ORs and 95% confidence intervals estimated using logistic regression and adjusted for sex, maternal allergy, SPT at 2 years (except for SPT model) and breastfeeding. Bold values denote statistical significance at the *p* < 0.05 level.

**TABLE 4:** Analysis of potential effect modification by endotoxin level, allergen skin prick test reactivity (SPT) at 2 years of age, maternal soil‐transmitted helminth infections (STH) and childhood STH infections during the first 2 years of life on associations between clinically significant respiratory disease (CSRD) subgroups and wheeze at 3 years of age.

CSRD sub‐group	Endotoxin		SPT at 2 years		Maternal geohelminths		Childhood geohelminths	
	High	Low	Yes	No	Yes	No	Yes	No
CSRD								
No	1	1	1	1	1	1	1	1
Yes								
RSV+	2.37 (0.39–14.55)	1.58 (0.15–15.77)	‐‐‐‐‐‐‐‐‐‐‐‐‐‐‐‐‐‐‐‐‐‐	4.00 (0.87–18.36)	0.57 (0.55–3.26)	5.46 (0.84–35.66)	‐‐‐‐‐‐‐‐‐‐‐‐‐‐‐‐‐‐‐‐‐‐	3.12 (0.71–13.69)
RHV+	**2.78 (1.01–7.66)**	2.16 (0.81–5.70)	**9.42 (1.88–47.15)**	1.92 (0.84–4.38)	1.50 (0.55–4.12)	**3.04 (1.12–8.26)**	**13.41 (1.56–115.64)**	1.68 (0.73–3.84)
RSV+/RHV+	**4.58 (1.25–18.11)**	3.17 (0.83–12.04)	6.75 (0.58–78.56)	3.00 (0.97–9.13)	2.27 (0.65–7.85)	4.19 (0.90–19.50)	**45.89 (2.78–757.66)**	2.27 (0.74–6.94)
RSV‐/RHV‐	1.95 (0.59–6.47)	2.30 (0.72–7.34)	2.14 (0.32–14.33)	1.93 (0.74–5.06)	1.08 (0.32–3.66)	2.87 (0.87–9.50)	1.77 (0.10–31.31)	2.38 (0.95–5.92)
No PCR	2.71 (0.84–8.77)	0.79 (0.15–4.25)	1.48 (0.12–18.19)	2.03 (0.74–5.55)	0.95 (0.27–3.37)	**4.19 (1.14–15.42)**	1.97 (0.11–35.84)	2.34 (0.86–6.36)
Any RSV+	**3.63 (1.08–12.19)**	2.69 (0.78–9.30)	2.52 (0.30–20.96)	**3.25 (1.20–8.76)**	1.71 (0.53–5.54)	**4.34 (1.16–16.14)**	**12.97 (1.08–156.47)**	2.37 (0.89–6.32)
Any RHV+	**3.11 (1.19–8.15)**	3.25 (0.93–5.92)	**8.86 (1.88–41.77)**	**2.18 (1.00–4.72)**	1.68 (0.65–4.34)	**3.19 (1.20–8.42)**	**14.51 (1.70–123.52)**	1.78 (0.82–3.84)

*Note*: Shown are ORs and 95% confidence intervals estimated using logistic regression and adjusted for sex, maternal allergy, SPT at 2 years (except for SPT model) and breastfeeding. Bold values denote statistical significance at the *p* < 0.05 level.

## 4 DISCUSSION

Here, we used a surveillance sample nested within a birth cohort to explore the effects of CSRD associated with viral infections during the first 2 years of life on wheeze/asthma to 8 years and airway function and inflammation at 8 years. The cohort was recruited in a low‐resource “pre‐industrial” setting in a rural tropical district of coastal Ecuador where infections with soil‐transmitted helminth parasites are endemic.^32^ We explored the effects of exposures that could affect airway inflammation during the first 2 years of life, namely in utero and early childhood exposures to STH parasites, SPT+, and endotoxin exposure in mattress dust. Our data provide evidence that CSRD, particularly when associated with RHV or mixed RHV/RSV infections, was associated with wheezing at 3 and 5 years. There is some evidence that early childhood atopy and post‐natal STH infections might enhance the effects of RHV on wheeze.

In this surveillance sample, the majority (67.7%) of children experienced CSRD during the first 2 years of life, substantial fractions of which were attributable to RSV (28%) or RHV (4.3%) infections. Wheezing illness was frequent before 8 years (≥18.0% at 3 and 5 years) but declined to below 6.3% by 8 years. CSRD was associated with recent and recurrent wheeze at 3 and 5 years, but the effects were not statistically significant at 8 years although strong positive associations persisted. These observations are consistent with what has been previously described in cohorts of children from high‐income settings such as Arizona, USA,^33^ and the Netherlands^34^, ^35^ in which LRIs are associated with early childhood wheeze, but with less clear effects on asthma at school‐age,^2^, ^33^, ^34^, ^35^ particularly for RSV. Rhinovirus has been more often associated with atopy and the development of recurrent wheeze and asthma at school age.^36^, ^37^ Consistent with a transient effect of virally induced CSRD on airway disease was the observation of no associations with diminished lung function, reversibility, and inflammation at 8 years, except for a positive association between RHV+CSRD and nasal eosinophilia.

Our findings showed associations between all‐cause CSRD (and, often, RHV+ CSRD) and recent or recurrent wheeze up to 5 years of age. In the case of RSV infections, trends of positive associations between RSV+ CSRD and wheeze symptoms were observed consistently, indicating a possible effect that this cohort had insufficient power to detect. Our ability to capture RSV in children before 6 months of age (and especially in children before 3 months of age) was limited by the timing of births among the children enrolled in respiratory surveillance relative to temporal patterns of RSV. Respiratory surveillance was not implemented for a significant proportion of the cohort prior to their first RSV season. Recent evidence, incompletely understood at the time of the study, indicates that RSV in Ecuador is strongly seasonal.^38^, ^39^ This likely underpowered our results, particularly for associations with RSV+ CSRD.

Historically, STH has been understood to downregulate Th2‐effector functions.^19^ However, *A. lumbricoides* is an STH that migrates through the lung during the larval stage of the life cycle and has been shown to induce strong and long‐lasting allergic inflammation in the airways of experimentally infected mice.^20^, ^21^ *A. lumbricoides* infection might therefore be expected to induce strong persistent Th2‐mediated inflammatory responses in the lungs of infected children, although real world data are lacking. A recent analysis from the same cohort provided evidence that children with greater *A. lumbricoides* parasite burdens at 5 years of age were more likely to have wheeze/asthma and elevated FeNO levels at 8 years.^17^ In this study, the associations between CSRD and wheeze at 3 years were stronger among children with early STH infections compared with uninfected children, although the interaction was not statistically significant. This effect appeared to be restricted to RHV+CSRD and could indicate a specific *A. lumbricoides*‐RHV interaction. There was no strong evidence that in utero exposure to STH infections (evaluated by STH infections in the last trimester of pregnancy) had strong effects on the associations between CSRD and pulmonary outcomes.

Atopy, measured using allergen skin prick test reactivity at 2 years, was used as an indicator of risk of allergic inflammation in the airways. There was evidence for a stronger association between CSRD and wheeze at 3 years among SPT+ compared to SPT‐ children although the interaction was not significant. Examining CSRD sub‐groups, it appeared that this effect was seen only for RHV+CSRD, while RSV+CSRD was less affected by atopy. These observations are consistent with the published literature on RHV+ and RSV+ LRTIs.^36^, ^37^ A “two‐hit” model has been proposed of a synergistic interaction between allergic sensitization and severe viral LRTIs in the development of childhood asthma.^4^ In the Childhood Origins of Asthma study, a US birth cohort of children at increased asthma risk, wheezing with RSV, RHV, or both viruses, was associated with an increased risk of asthma at 6 years of age^40^, while in an Australian high‐risk birth cohort, infant wheezing with RHV or RSV was a risk factor for current wheeze at 5 years.^41^ These studies showed that the risk of asthma is increased by severe LRTIs, particularly in the presence of atopy.^41^ There appears to be a specific interaction between early childhood allergic sensitization and RHV+ wheeze: RHV is a risk factor for the development of atopic asthma, whereas RSV has been associated with non‐atopic asthma.^3^

This study is subject to several limitations. The ECUAVIDA Cohort was not originally designed to study early life respiratory viral infections and NP swab collection was initiated in mid‐2009. The sample size for active surveillance was restricted by logistical and cost considerations. Because children were not enrolled into the cohort at a uniform rate and RSV is strongly seasonal in Ecuador,^38^, ^39^ a fact not appreciated at the time of the study, a minority of children younger than 6 months of age were under surveillance during the RSV season, possibly resulting in much of RSV disease in early infancy being missed. This could have introduced substantial bias, as children categorized as never having had RSV+ CSRD in the first 2 years of life may indeed have had such undocumented illnesses in 2009. Such bias would likely underestimate associations between RSV+CSRD and respiratory outcomes. In contrast to RSV, RHV was detected year‐round. High study retention (>85% until 8 years) should have minimized potential selection bias and detailed information collected on a wide variety of potential confounding factors should have limited uncontrolled confounding. We used CSRD to identify outpatient illnesses of the lower respiratory tract, given that hospitalization rates for lower respiratory illness would be expected to be low in a surveillance cohort of this size. CSRD likely represents a medically significant but non‐severe illness of the lower respiratory tract.

In conclusion, our data from a population of children followed prospectively in a low‐resource non‐industrialized setting in Latin America showed that CSRD during the first 2 years of life, particularly when associated with RHV infection or RHV/RSV co‐infection, was significantly associated with wheezing illness up to 5 years of age. Associations of early childhood RHV+CSRD and wheezing illness in later childhood were strongest in the presence of atopy and *A. lumbricoides* infections, both of which cause Th2‐mediated inflammation, indicating that Th2 inflammation in the lungs may contribute to severity of LRIs associated with RHV. Our findings are likely to be generalizable to similar non‐affluent pediatric populations living in rural tropical regions of Latin America. Larger prospective studies in similar STH‐endemic settings are required to better understand the interactions between *A. lumbricoides* and RHV and their long‐term effects on lung health.

## AUTHOR CONTRIBUTIONS

**Jessica Atwell**: Data curation (supporting); Formal analysis (supporting); Investigation (supporting); Writing – original draft (equal). **Martha Chico**: Data curation (lead); Investigation (supporting); Methodology (supporting); Project administration (equal); Supervision (equal); Writing – review & editing (supporting). **Maritza Vaca**: Investigation (supporting); Writing – review & editing (supporting). **Andrea Arevalo‐Cortes**: Methodology (supporting); Writing – review & editing (supporting). **Ruth Karron**: Conceptualization (equal); Funding acquisition (equal); Resources (supporting); Supervision (equal); Writing – review & editing (supporting). **Philip J. Cooper**: Conceptualization (equal); Formal analysis (lead); Funding acquisition (equal); Investigation (equal); Methodology (equal); Project administration (equal); Resources (lead); Supervision (equal); Writing – original draft (equal).

## CONFLICT OF INTEREST STATEMENT

The authors declare that they have no conflicts of interest.

## Supporting information

Supporting Information S1

## Data Availability

The data that support the findings of this study are available from the corresponding author upon reasonable request.

## References

[1] Li Y , Wang X , Blau DM , et al. Global, regional, and national disease burden estimates of acute lower respiratory infections due to respiratory syncytial virus in children younger than 5 years in 2019: a systematic analysis. Lancet. 2022;399(10340):2047‐2064. 10.1016/s0140-6736(22)00478-0 35598608 PMC7613574

[2] Meissner HC . Viral bronchiolitis in children. N Engl J Med. 2016;374(1):62‐72. 10.1056/nejmra1413456 26735994

[3] Mikhail I , Grayson MH . Asthma and viral infections: an intricate relationship. Ann Allergy Asthma Immunol. 2019;123(4):352‐358. 10.1016/j.anai.2019.06.020 31276807 PMC7111180

[4] Ahanchian H , Jones CM , Chen YS , Sly PD . Respiratory viral infections in children with asthma: do they matter and can we prevent them? BMC Pediatr. 2012;12(1):147. 10.1186/1471-2431-12-147 22974166 PMC3471019

[5] Esteban I , Stein RT , Polack FP . A durable relationship: respiratory syncytial virus bronchiolitis and asthma past their golden anniversary. Vaccines. 2020;8(2):201. 10.3390/vaccines8020201 32357557 PMC7350256

[6] Brunwasser SM , Snyder BM , Driscoll AJ , et al. Assessing the strength of evidence for a causal effect of respiratory syncytial virus lower respiratory tract infections on subsequent wheezing illness: a systematic review and meta‐analysis. Lancet Respir Med. 2020;8:795‐806. 10.1016/s2213-2600(20)30109-0 32763206 PMC7464591

[7] Wu P , Hartert TV . Evidence for a causal relationship between respiratory syncytial virus infection and asthma. Expert Rev Anti Infect Ther. 2011;9:731‐745. 10.1586/eri.11.92 21905783 PMC3215509

[8] Jartti T , Gern JE . Role of viral infections in the development and exacerbation of asthma in children. J Allergy Clin Immunol. 2017;140(4):895‐906. 10.1016/j.jaci.2017.08.003 28987219 PMC7172811

[9] Pullan CR , Hey EN . Wheezing, asthma, and pulmonary dysfunction 10 years after infection with respiratory syncytial virus in infancy. Br Med J. 1982;284(6330):1665‐1669. 10.1136/bmj.284.6330.1665 6805648 PMC1498624

[10] Murray M , Webb MS , O'Callaghan C , Swarbrick AS , Milner AD . Respiratory status and allergy after bronchiolitis. Arch Dis Child. 1992;67(4):482‐487. 10.1136/adc.67.4.482 1580676 PMC1793348

[11] Noble V , Murray M , Webb MS , Alexander J , Swarbrick AS , Milner AD . Respiratory status and allergy nine to 10 years after acute bronchiolitis. Arch Dis Child. 1997;76(4):315‐319. 10.1136/adc.76.4.315 9166022 PMC1717138

[12] Sigurs N , Gustafsson PM , Bjarnason R , et al. Severe respiratory syncytial virus bronchiolitis in infancy and asthma and allergy at age 13. Am J Respir Crit Care Med. 2005;171(2):137‐141. 10.1164/rccm.200406-730oc 15516534

[13] Strachan DP . Family size, infection and atopy: the first decade of the “hygiene hypothesis”. Thorax. 2000;55(90001):S2‐S10. 10.1136/thorax.55.suppl_1.s2 10943631 PMC1765943

[14] Pfefferle PI , Keber CU , Cohen RM , Garn H . The hygiene hypothesis ‐ learning from but not living in the past. Front Immunol. 2021;12:635935. 10.3389/fimmu.2021.635935 33796103 PMC8007786

[15] Rodrigues LC , Newcombe PJ , Cunha SS , et al. Early infection with Trichuris trichiura and allergen skin test reactivity in later childhood. Clin Exp Allergy. 2008;38(0):1769‐1777. 10.1111/j.1365-2222.2008.03027.x 18547322

[16] Caraballo L , Zakzuk J , Lee BW , et al. Particularities of allergy in the tropics. World Allergy Organ J. 2016;9:20. 10.1186/s40413-016-0110-7 27386040 PMC4924335

[17] Cooper PJ , Chis Ster I , Chico ME , et al. Impact of early life geohelminths on wheeze, asthma and atopy in Ecuadorian children at 8 years. Allergy. 2021;76(9):2765‐2775. 10.1111/all.14821 33745189 PMC8496980

[18] Arrais M , Maricoto T , Nwaru BI , et al. Helminth infections and allergic diseases: systematic review and meta‐analysis of the global literature. J Allergy Clin Immunol. 2022;149(6):2139‐2152. 10.1016/j.jaci.2021.12.777 34968529

[19] Else KJ , Keiser J , Holland CV , et al. Whipworm and roundworm infections. Nat Rev Dis Prim. 2020;6(1):44. 10.1038/s41572-020-0171-3 32467581

[20] Weatherhead JE , Porter P , Coffey A , et al. Ascaris larval infection and lung invasion directly induce severe allergic airway disease in mice. Infect Immun. 2018;86(12):e005333‐18. 10.1128/iai.00533-18 PMC6246907 30249744

[21] Wu Y , Li E , Knight M , et al. Transient Ascaris suum larval migration induces intractable chronic pulmonary disease and anemia in mice. PLoS Negl Trop Dis. 2021;15(12):e0010050. 10.1371/journal.pntd.0010050 34914687 PMC8717995

[22] Jõgi NO , Kitaba N , Storaas T , et al. Ascaris exposure and its association with lung function, asthma, and DNA methylation in Northern Europe. J Allergy Clin Immunol. 2022;149(6):1960‐1969. 10.1016/j.jaci.2021.11.013 34996616

[23] Gelpi AP , Mustafa A . Ascaris pneumonia. Am J Med. 1968;44(3):377‐389. 10.1016/0002-9343(68)90109-5 5641301

[24] Cooper PJ , Chico ME , Platts‐Mills TA , Rodrigues LC , Strachan DP , Barreto ML . Cohort profile: the Ecuador life (ECUAVIDA) study in Esmeraldas Province, Ecuador. Int J Epidemiol. 2015;44(5):1517‐1527. 10.1093/ije/dyu128 24990475 PMC4681103

[25] Cooper PJ , Ster IC , Chico ME , Vaca M , Barreto ML , Strachan DP . Patterns of allergic sensitization and factors associated with emergence of sensitization in the rural tropics early in the life course: findings of an Ecuadorian birth cohort. Front Allergy. 2021;2:687073. 34888545 10.3389/falgy.2021.687073 PMC7612078

[26] Quanjer PH , Stanojevic S , Cole TJ , et al. Multi‐ethnic reference values for spirometry for the 3‐95‐yr age range: the global lung function 2012 equations. Eur Respir J. 2012;40(6):1324‐1343. 10.1183/09031936.00080312 22743675 PMC3786581

[27] Dweik RA , Boggs PB , Erzurum SC , et al. An official ATS clinical practice guideline: interpretation of exhaled nitricoxide levels (FENO) for clinical applications. Am J Respir Crit Care Med. 2011;184(5):602‐615. 10.1164/rccm.9120-11st 21885636 PMC4408724

[28] Ardura‐Garcia C , Arias E , Hurtado P , et al. Predictors of severe asthma attack re‐attendance in Ecuadorian children: a cohort study. Eur Respir J. 2019;54(5):1802419. 10.1183/13993003.02419-2018 31515399 PMC6860994

[29] Marinho S , Simpson A , Lowe L , Kissen P , Murray C , Custovic A . Rhinoconjunctivitis in 5‐year‐old children: a population‐based birth cohort study. Allergy. 2007;62(4):385‐393. 10.1111/j.1398-9995.2006.01294.x 17362249

[30] Taniuchi M , Islam K , Sayeed MA , et al. Etiology of diarrhea requiring hospitalization in Bangladesh by quantitative polymerase chain reaction, 2014‐2018. Clin Infect Dis. 2021;73(9):e2493‐e2499. 10.1093/cid/ciaa840 32592580 PMC8563176

[31] Calvopiña M . Terapéutica Antiparasitaria. 2nd ed. Ministerio de Salud Pública del Ecuador; 1997.

[32] Chis Ster I , Niaz HF , Chico ME , Oviedo Y , Vaca M , Cooper PJ . The epidemiology of soil‐transmitted helminth infections in children up to 8 years of age: findings from an Ecuadorian birth cohort. PLoS Negl Trop Dis. 2021;15(11):e0009972. 10.1371/journal.pntd.0009972 34797823 PMC8641893

[33] Stein RT , Sherrill D , Morgan WJ , et al. Respiratory syncytial virus in early life and risk of wheeze and allergy by age 13 years. Lancet. 1999;354(9178):541‐545. 10.1016/s0140-6736(98)10321-5 10470697

[34] Blanken MO , Rovers MM , Molenaar JM , et al. Respiratory syncytial virus and recurrent wheeze in healthy preterm infants. N Engl J Med. 2013;368(19):1791‐1799. 10.1056/nejmoa1211917 23656644

[35] Scheltema NM , Nibbelke EE , Pouw J , et al. Respiratory syncytial virus prevention and asthma in healthy preterm infants: a randomised controlled trial. Lancet Respir Med. 2018;6(4):257‐264. 10.1016/s2213-2600(18)30055-9 29500030

[36] Robinson PFM , Fontanella S , Ananth S , et al. Recurrent severe preschool wheeze: from prespecified diagnostic labels to underlying endotypes. Am J Respir Crit Care Med. 2021;204(5):523‐535. 10.1164/rccm.202009-3696oc 33961755 PMC8491264

[37] Makrinioti H , Hasegawa K , Lakoumentas J , et al. The role of respiratory syncytial virus‐ and rhinovirus‐induced bronchiolitis in recurrent wheeze and asthma ‐ a systematic review and meta‐analysis. Pediatr Allergy Immunol. 2022;33(3):e13741. 10.1111/pai.13741 35338734

[38] Jonnalagadda S , Rodriguez O , Estrella B , Sabin LL , Sempertegui F , Hamer DH . Etiology of severe pneumonia in Ecuadorian children. PLoS One. 2017;12(2):e0171687. 10.1371/journal.pone.0171687 28182741 PMC5300242

[39] Azziz‐Baumgartner E , Bruno A , Daugherty M , et al. Incidence and seasonality of respiratory viruses among medically attended children with acute respiratory infections in an Ecuador birth cohort, 2011‐2014. Influenza Other Respir Viruses. 2022;16(1):24‐33. 10.1111/irv.12887 34432362 PMC8692806

[40] Jackson DJ , Gangnon RE , Evans MD , et al. Wheezing rhinovirus illnesses in early life predict asthma development in high‐risk children. Am J Respir Crit Care Med. 2008;178(7):667‐672. 10.1164/rccm.200802-309oc 18565953 PMC2556448

[41] Kusel MM , de Klerk NH , Kebadze T , et al. Early‐life respiratory viral infections, atopic sensitization, and risk of subsequent development of persistent asthma. J Allergy Clin Immunol. 2007;119(5):1105‐1110. 10.1016/j.jaci.2006.12.669 17353039 PMC7125611

