# Supplementary material for: Effect of infant viral respiratory disease on childhood asthma in a non‐industrialized setting
Source: Clin Transl Allergy. 2023 Aug 8;13(8):e12291. doi: 10.1002/clt2.12291 (PMC10408584; doi:10.1002/clt2.12291)
Supplement: Supplementary file 1 — Supporting Information S1 [file CLT2-13-e12291-s001.docx]

| **CSRD group** | **N (%)** | **Rhinoconjunctivitis at 3 years**  **(n=401)** | | | **Rhinoconjunctivitis at 5 years**  **(n=380)** | | | **Rhinoconjunctivitis at 8 years**  **(n=363)** | | |
| --- | --- | --- | --- | --- | --- | --- | --- | --- | --- | --- |
|  |  | **N(%)** | **OR (95% CI)** | **P value** | **N(%)** | **OR (95% CI)** | **P value** | **N(%)** | **OR (95% CI)** | **P value** |
| CSRD to 2 years  No  Yes  All  RSV+  RHV+  RSV+/RHV+  RSV-RHV-  No PCR result | 137 (32.3)  287 (67.7)  18 (4.3)  119 (28.0)  32 (7.5)  63 (14.9)  55 (13.0) | 18 (14.0)  32 (11.8)  2 (12.5)  8 (7.0)  6 (20.0)  8 (13.1)  8 (16.0) | 1  0.82 (0.44-1.53)  0.88 (0.18-4.21)  0.46 (0.19-1.11)  1.54 (0.55-4.29)  0.93 (0.38-2.28)  1.17 (0.48-2.90) | 0.536  0.874  0.083  0.407  0.875  0.728 | 17 (13.8)  38 (14.8)  1 (6.3)  16 (14.8)  3 (10.3)  7 (12.5)  11 (22.9) | 1  1.08 (0.58-2.01)  0.42 (0.05-3.35)  1.08 (0.52-2.27)  0.72 (0.20-2.64)  0.89 (0.35-2.29)  1.85 (0.80-4.32) | 0.803  0.410  0.830  0.620  0.810  0.153 | 8 (7.0)  25 (10.1)  1 (7.7)  12 (11.2)  2 (8.0)  2 (3.6)  8 (17.0) | 1  1.50 (0.65-3.43)  1.11 (0.13-9.69)  1.69 (0.66-4.31)  1.16 (0.23-5.84)  0.50 (0.10-2.41)  2.74 (0.96-7.81) | 0.338  0.922  0.272  0.854  0.385  0.060 |

Supplementary Table 1. Univariate associations between clinically significant respiratory disease (CSRD) and childhood rhinoconjunctivitis between 3 and 8 years of age.

ORs and 95% confidence intervals (CI) estimated using logistic regression. Separate models were used to estimate associations with all CSRD and CSRD by respiratory virus subgroups. RSV – PCR+ for respiratory syncytial virus. RHV+ - PCR+ for rhinovirus; RSV+/RHV+ - PCR+ for both viruses; RSV-/RHV- - PCR- for both viruses.
